# Path Forward to Optimise Post-approval Change Management and Facilitate Continuous Supply of Medicines and Vaccines of High Quality Worldwide: Joint Position from EFPIA, IFPMA and Vaccines Europe

**DOI:** 10.1007/s43441-022-00426-9

**Published:** 2022-08-02

**Authors:** Andrew Deavin, Sarah Adam, Susanne Ausborn, Ane Sofie Böhm Nielsen, Sonia Cappellini, Isabelle Colmagne-Poulard, Thierry Gastineau, Arturo Gonzalez-Martinez, Sylvie Meillerais, Charlie Mortazavi

**Affiliations:** 1 GSK, 20 Avenue Fleming, 1300 Wavre, Belgium; 2 The International Federation of Pharmaceutical Manufacturers and Associations, Chemin des Mines 9, 1202 Geneva, Switzerland; 3 F. Hoffmann - La Roche Ltd, Grenzacher Strasse 124, 4070 Basel, Switzerland; 4 Novo Nordisk, Vandtaarnsvej 108, 2860 Soeborg, Denmark; 5 Menarini Ricerche S.p.A, Via Tito Speri 10, Pomezia, Rome, Italy; 6 Merck KGaA, Chemin de l’Ouriettaz, 1170 Aubonne, Switzerland; 7 Sanofi, 14 Espace Henry Vallée, 69007 Lyon, France; 8 MSD-Europe Inc., Clos du Lynx, 5, Lynx Binnenhof, 1200 Brussels, Belgium; 9 Sanofi R&D, 1 Avenue Brossolette, 91385 Chilly Mazarin, France

**Keywords:** Post-approval change management, Reliance, ICH Q12, Supply, Medicines and vaccines

## Abstract

Post-approval changes (PACs) to the registered information of authorised medicinal products are introduced routinely worldwide to enhance the robustness and efficiency of the manufacturing process, ensure timely supply in case of increased demand, improve quality control techniques, respond to changes in regulatory requirements and upgrade to state-of-the-art facilities. These are critical to prevent supply disruption and continuously improve existing medicines and vaccines. Due to the complexity of current PAC systems across markets, a change can take 3 to 5 years to approval globally (Hoath et al in BioProcess Int, 2016) thus hindering innovation and increasing the risk of shortages. The key messages are as follows: 1. Industry believes that global regulatory convergence of post-approval changes to Marketing Authorisations (MAs) using science- and risk-based approaches will enable a more efficient management of quality and supply improvements and will facilitate patients’ access to innovative medicines and vaccines of the highest quality. 2. National Regulatory Authorities (NRAs) should establish national or regional guidelines in line with international standards (regarding a risk-based classification of changes and standardisation of requirements) (Guidelines on procedures and data requirements for changes to approved biotherapeutic products, in WHO Technical Report Series, 2018, Guidelines on procedures and data requirements for changes to approved vaccines, in WHO Technical Report Series, 2015), have clear procedural guidance including timelines and implement reliance pathways to accelerate the approval of changes. This paper briefly outlines the challenges for PACs and provides solutions for a more flexible and aligned global system.

## Introduction

Post-approval changes (PACs) to the registered information of authorised medicinal products are introduced routinely worldwide to enhance the robustness and efficiency of the manufacturing process, ensure timely supply in case of increased demand, improve quality control techniques, respond to changes in regulatory requirements and upgrade to state-of-the-art facilities. This continued effort is critical to prevent supply disruption and continuously improve existing medicines and vaccines and is, in many ways, as important as bringing new medicines and vaccines to the market. It is also important when the product development and registration process is accelerated to meet unmet medical needs, thus pushing changes that would typically be made during development into the post-approval setting.

**Fig. 1:**
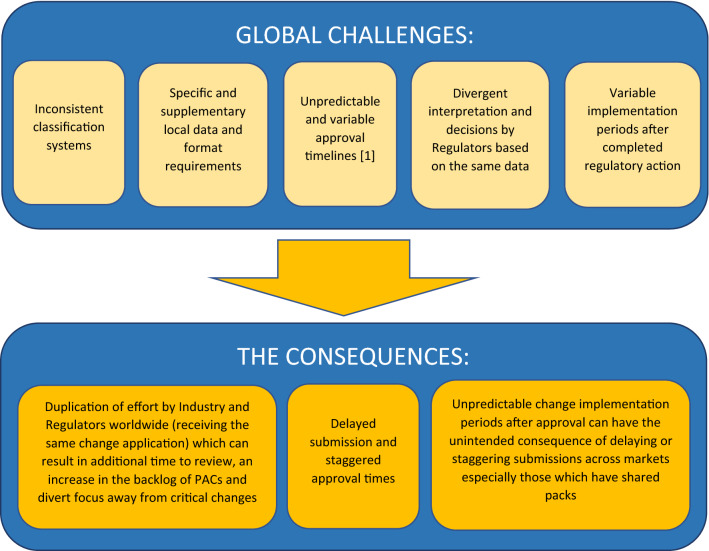
Challenges of the current global regulatory landscape for PACs. This figure illustrates the challenges that are currently observed when managing PACs and the subsequent consequences that are experienced by the industry

The challenges and consequences of the current global regulatory landscape for PACs are shown in Fig. 1.

## Tools for Efficient PACs Management

### Use of Reliance Practices Should Be Maximised

Collaboration amongst Regulators has been shown to be essential to make better use of available resources and to enable more efficient regulatory pathways leading to fast access of medicines and vaccines to patients [4].

Industry strongly supports the International Coalition of Medicines Regulatory Authorities (ICMRA), International Conference of Drug Regulatory Authorities (ICDRA) and World Health Organisation (WHO) positions on the use of reliance [4–6] and its broader application throughout the full lifecycle of a product, including for GMP inspections and lot release [7, 8]. Of note is that if a Certificate of Pharmaceutical Product (CPP) is required by the NRA, then reliance should be applied (without further data requirements), and the review shortened.

### Requirements for the Submission of PACs Should Be Converged

Data requirements for PACs should be adapted to the risk level and limited to those that are scientifically justifiable. We propose that data which can be verified during inspection should be eliminated. Industry encourages the adoption of the International Council on Harmonisation (ICH)—Common Technical Document format/content for a harmonised dossier throughout the lifecycle to facilitate the preparation and submission process.

### PAC Classification and Timelines Should Be Converged and Common Risk-Based Approaches Adopted

A common regulatory understanding of risk-based approaches and risk-based classification of changes is essential for post-approval changes management as highlighted in the ICH Q12 [9] and World Health Organisation (WHO) guidelines on procedures and data requirements for changes [2, 3]. To provide a further breakdown of specific changes, data requirements and timelines, we recommend alignment with the WHO guidelines [2, 3, 10].

Maximum review periods for changes should be established as recommended in the WHO guidance [3]: major changes, a maximum of 6 months review; moderate changes, a maximum of 3 months review; and minor changes, only requiring a notification to the NRA.

Finally building on the concept of reliance and risk-based approaches, the following should be implemented:

- Changes with no impact on quality, safety or efficacy should be managed internally within companies’ Pharmaceutical Quality System (PQS) without any reporting to NRAs as per ICH Q12 [11]
- When already reviewed and approved in a reference country, a major or moderate change should be accelerated in the relying country via a verification or abridged pathway (i.e. a risk-based approach).

### The Use of Tools in ICH Q12 (Technical and Regulatory Considerations for Pharmaceutical Product Lifecycle Management) Should Be Maximised [9]

The implementation of ICH Q12 introduces several important tools and related concepts which we believe can help to streamline technical change management based on process understanding and risk-based approaches [12].

To determine whether to prioritise implementation of ICH Q12 tools, we recommend to first consider the level of maturity of the regulatory framework for PACs that is in place.

For rapid benefit of ICH Q12, implementation of the post-approval change management protocol (PACMP) is recommended. This tool can allow an appropriate lower reporting category to enable the reporting of results after executing the protocol as agreed with the Health Authorities.

### Flexible Market Implementation to Ensure Supply Continuity

Pre- and post-change product versions should co-exist in the market during a transition period. Flexible implementation timelines are needed to ensure smooth transition and supply continuity of the post-change product version, whilst approval processes may still be on-going in other regions of the world. This is especially important to accommodate the long lag time needed to supply global products with complex manufacturing processes to reduce potential supply chain disruption.

### Emergency Preparedness Considerations

**Fig. 2:**
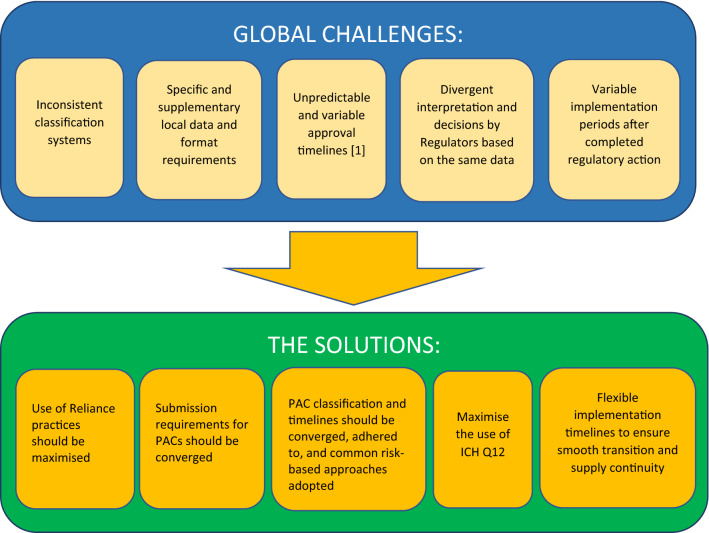
Solutions to the Current Global Regulatory Landscape for PACs. This figure illustrates the solutions that could help to resolve the challenges currently faced by industry when managing PACs

The recent Covid-19 pandemic has led to a re-think in terms of how changes should be managed especially in an emergency [13]. Regulators and the pharmaceutical Industry have made significant efforts to mitigate issues and to find innovative approaches to accelerate PACs. One example is a pilot for collaborative assessment of post-approval change mechanisms [14]. Such collaborative efforts should be continued (Fig. 2).

We believe the following elements are particularly critical for major changes applied to therapeutics or vaccines, such as addition of new manufacturing sites in the context of technology transfer to expand manufacturing capacity in a short timeframe:

- PAC review periods for emergency applications should be accelerated, with suitable timelines given.
- Transparent communication and coordinated dialogue amongst stakeholders are critical elements for success.
- Rather than having multiple reference authorities in an emergency procedure, consideration should be given to limit to the first market where the relevant change package has been submitted and approved by a reference authority.

## Conclusion

Regulatory oversight is critical to ensure that high-quality and effective medicines and vaccines are available in a country. Regulators and the pharmaceutical Industry have a collective responsibility to assure an uninterrupted supply of compliant, safe and efficacious medicines and vaccines to patients globally.

Regulators as well as Industry believe that the regulatory process for managing post-approval changes needs to be significantly simplified to facilitate global supply of medicines and vaccines. This can be achieved with consistent, harmonised and clear classifications and adherence to timelines, increased reliance between Regulators and the use of novel regulatory and scientific tools. International collaboration and cooperation towards regulatory convergence are a good way to address the increasing workload challenges of NRAs [15].

We acknowledge that further effort needs to be made by the Industry to reduce the complexity of managing post-approval changes. These measures include advanced planning of changes at start of the lifecycle, more strategic combination of changes as well as transparent communication of supply challenges to Regulators.

We believe action by all stakeholders is required to develop an efficient change management system that contributes to enhancing global public health by providing equitable and timely access to medicines and vaccines.
